# Reversible bacterial immobilization based on the salt-dependent adhesion of the bacterionanofiber protein AtaA

**DOI:** 10.1186/s12934-017-0740-7

**Published:** 2017-07-18

**Authors:** Shogo Yoshimoto, Yuki Ohara, Hajime Nakatani, Katsutoshi Hori

**Affiliations:** Department of Biomolecular Engineering, Graduate School of Engineering, Nagoya University, Furo-cho, Chikusa-ku, Nagoya, 464-8603 Japan

**Keywords:** Immobilization, Whole-cell catalyst, Trimeric autotransporter adhesin, *Acinetobacter*

## Abstract

**Background:**

Immobilization of microbial cells is an important strategy for the efficient use of whole-cell catalysts because it simplifies product separation, enables the cell concentration to be increased, stabilizes enzymatic activity, and permits repeated or continuous biocatalyst use. However, conventional immobilization methods have practical limitations, such as limited mass transfer in the inner part of a gel, gel fragility, cell leakage from the support matrix, and adverse effects on cell viability and catalytic activity. We previously showed a new method for bacterial cell immobilization using AtaA, a member of the trimeric autotransporter adhesin family found in *Acinetobacter* sp. Tol 5. This approach is expected to solve the drawbacks of conventional immobilization methods. However, similar to all other immobilization methods, the use of support materials increases the cost of bioprocesses and subsequent waste materials.

**Results:**

We found that the stickiness of the AtaA molecule isolated from Tol 5 cells is drastically diminished at ionic strengths lower than 10 mM and that it cannot adhere in deionized water, which also inhibits cell adhesion mediated by AtaA. Cells immobilized on well plates and polyurethane foam in a salt solution were detached in deionized water by rinsing and shaking, respectively. The detached cells regained their adhesiveness in a salt solution and could rapidly be re-immobilized. The cells expressing the *ataA* gene maintained their adhesiveness throughout four repeated immobilization and detachment cycles and could be repeatedly immobilized to polyurethane foam by a 10-min shake in a flask. We also demonstrated that both bacterial cells and a support used in a reaction could be reused for a different type of reaction after detachment of the initially immobilized cells from the support and a subsequent immobilization step.

**Conclusions:**

We invented a unique reversible immobilization method based on the salt-dependent adhesion of the AtaA molecule that allows us to reuse bacterial cells and supports by a simple manipulation involving a deionized water wash. This mitigates problems caused by the use of support materials and greatly helps to enhance the efficiency and productivity of microbial production processes.

**Electronic supplementary material:**

The online version of this article (doi:10.1186/s12934-017-0740-7) contains supplementary material, which is available to authorized users.

## Background

Although microbial cells are expected to provide environmentally friendly production processes as whole-cell biocatalysts with highly effective and selective reactivity under ordinary temperatures and pressures [1–5], their disadvantages, such as complicated handling due to their fragility, a requirement for costly product separation processes, and easy inactivation at unsuitable temperatures, pH, and substrate and product concentrations, raise production costs and hinder their commercial use in chemical production processes. To expand the use of microbial production processes in the industrial sector, it is important to improve the efficiency of the entire process, from an upstream process including strain development to a downstream process including product separation [6]. Recently, a systems metabolic engineering approach targeting an upstream process received considerable attention by aiming to develop a novel biosynthetic pathway producing high-value products and/or improve their productivity in microbial cells [6–11]. As for the downstream process, cell immobilization is important because it simplifies product separation, enables the cell concentration to be increased, stabilizes the enzymatic activity, and permits repetitive or continuous use of precious and expensive biocatalysts [12–15]. Conventional methods for cell immobilization are gel entrapment, covalent bonding to solid surfaces, cross-linkage, and physical adsorption [16, 17]. These methods, however, have practical limitations, such as limited mass transfer in the inner part of a gel [18, 19], gel fragility, cell leakage from the support matrix, and adverse effects on cell viability and catalytic activity [12].

We previously invented a method for bacterial cell immobilization using the adhesive protein AtaA found in *Acinetobacter* sp. Tol 5 [20–22], which belongs to the trimeric autotransporter adhesin (TAA) family [23]. Although AtaA shares a fibrous architecture consisting of an N-terminus—passenger domain (PSD) containing head and stalk domains—transmembrane anchor (TM)—C-terminus with TAA family members [24], which usually bind to target biotic surfaces, AtaA uniquely confers nonspecific high adhesiveness to both abiotic and biotic surfaces on bacterial cells transformed with its gene. Large amounts of growing, resting, even lyophilized transformant cells can be quickly and firmly immobilized onto any material surfaces selected according to the application [25]. Cells immobilized directly on surfaces through AtaA are not embedded in extracellular polymeric substances with mass transfer limitations, show enhanced tolerance [22], increase chemical reaction rates, and can be repeatedly used in reactions without inactivation [25]. However, similar to all other immobilization methods, the use of support materials increases the cost of bioprocesses and subsequent waste materials. These might be inevitable problems as long as support materials are used in the immobilization process. A way to minimize these drawbacks should be developed so as to, for example, reduce the amount of support materials, use inexpensive materials or waste materials, and reuse support materials.

AtaA is a homotrimer of polypeptides comprising 3630 amino acids. In a previous study, we developed a method to isolate its PSD, which is secreted to the bacterial cell surface through the TM and is responsible for biological functions, by genetically introducing a recognition site for human rhinovirus 3C (HRV 3C) protease [26]. Specific cleavage by the protease reaps AtaA PSD nanofibers 225 nm in length from the cell surface. This enables biochemical and biophysical analyses of the purified huge AtaA PSD in the native molecular state. Here, we demonstrate a new phenomenon: AtaA PSD cannot adhere to surfaces in deionized water (dH_2_O). Based on this molecular property of AtaA, we developed a unique method for the reversible immobilization of bacterial cells, which can solve the problems caused by the use of support materials.

## Results

### Effect of ionic strength on the adhesive property of the AtaA molecule

**Fig. 1:**
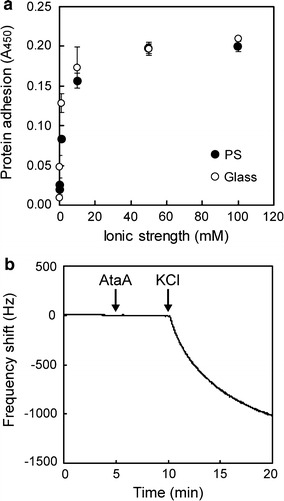
Effect of ionic strength on the adhesion of purified AtaA PSD. **a** Adhesion of AtaA PSD as a function of ionic strength. Amounts of AtaA PSD adhered to wells of a polystyrene (PS) or a glass 96-well plate were quantified by ELISA with anti-AtaA antiserum. Data are expressed as mean ± SEM (n = 3). **b** Ion-dependent adhesion of AtaA PSD was analyzed using QCM. *Arrows* indicate the addition of AtaA PSD dissolved in dH_2_O and KCl solution

To investigate the adhesive property of the AtaA molecule, AtaA PSD was isolated by the enzymatic reaping method from a Tol 5 derivative strain, 4140, transformed with p3CAtaA [26, 27]. KCl solutions (50 µL) of various concentrations containing 5 μg/mL of the purified AtaA PSD were incubated in 96-well polystyrene (PS) and glass plates at 28 °C for 2 h. The protein solution was removed from each well using a micropipette and the well was rinsed three times with 200 μL of phosphate-buffered saline containing 0.05% Tween-20 (PBS-T). The AtaA PSD adsorbed to the well plates was assessed by ELISA. Interestingly, we found that the amounts of AtaA PSD molecules adsorbed onto surfaces of hydrophobic PS and hydrophilic glass dropped sharply at ionic strengths lower than 10 mM, with the molecules hardly adhering in dH_2_O, despite their high adhesiveness at higher ionic strengths (Fig. 1a). AtaA PSD cannot be considered to be denatured in dH_2_O because AtaA PSD has high structural stability [26, 28]. Indeed, AtaA PSD molecules isolated from cells by the enzymatic reaping method mentioned above were dissolved in dH_2_O, and subsequently KCl solution was added to the adherence assay to attain the final ionic strengths intended. Furthermore, by using a quartz crystal microbalance (QCM), which enables the quantification of molecules adhered to its quartz crystal sensor chip as a frequency shift, we confirmed that the adhesiveness of AtaA PSD can be recovered in a salt solution by adding KCl salt to fresh water. AtaA PSD molecules did not adhere to a gold-coated sensor chip of QCM in dH_2_O, but started adhering to the chip immediately after KCl solution was added (Fig. 1b). Evidently, AtaA PSD is not denatured in dH_2_O and recovers its adhesiveness in a salt solution.

### Effect of the ionic strength on bacterial cell adhesion mediated by AtaA

**Fig. 2:**
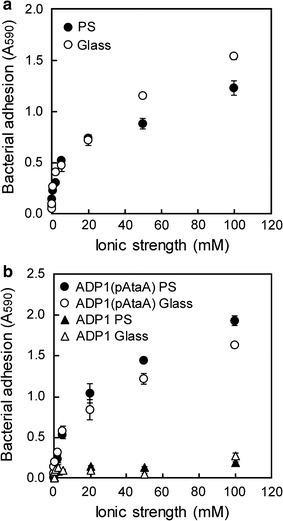
Effect of ionic strength on the adhesion of bacterial cells to material surfaces. **a** Adhesion of Tol 5 cells as a function of ionic strength. Tol 5 cells adhering to wells of a PS or a glass 96-well plate were quantified by crystal violet staining. **b** Adhesion of ADP1 and derivative cells as a function of ionic strength. ADP1 wild-type and ADP1 (pAtaA) cells adhering to the wells of a PS or a glass 96-well plate were quantified by crystal violet staining. Data are expressed as mean ± SEM (n = 3)

The identification of the ionic strength-dependent stickiness of the AtaA PSD prompted us to examine whether or not bacterial cell adhesion mediated by AtaA also depends on ionic strength. Tol 5 cells were grown, harvested, washed with dH_2_O, suspended in dH_2_O and KCl solutions of various concentrations at an OD_660_ of 0.5, and placed into 96-well plates. After a 2-h incubation at 28 °C without shaking, the cell suspension was removed from each well by a micropipette and the well was rinsed three times with 200 μL of dH_2_O or KCl solution of each concentration using a micropipette. The cells immobilized onto the well surfaces were quantified by crystal violet staining. At ionic strengths higher than 20 mM, a large amount of Tol 5 cells was immobilized onto PS and glass surfaces, although the amount gradually increased as the ionic strength increased (Fig. 2a). However, Tol 5 cell adhesion dropped at ionic strengths lower than 5 mM for both PS and glass surfaces, and Tol 5 cells were unable to adhere to either surface in dH_2_O. To confirm that such ionic strength-dependent adhesion of bacterial cells can be decisively attributed to the adhesive properties of the AtaA molecule, the cell adhesion of *Acinetobacter baylyi* ADP1 and its transformant with *ataA*, ADP1 (pAtaA), to PS and glass surfaces was examined at various ionic strengths by the same procedure used for Tol 5 cells. ADP1 cells expressing AtaA showed high adhesiveness to both PS and glass surfaces at ionic strengths higher than 20 mM with a gradual increase in adhesion with ionic strength, whereas wild-type ADP1 was hardly immobilized at any ionic strength (Fig. 2b). However, at an ionic strength lower than 5 mM, even ADP1 cells expressing AtaA showed the same diminished adhesion as Tol 5 cells, adhering to neither PS surface nor glass surface in dH_2_O. Therefore, the adhesion profiles of Tol 5 cells and ADP1 (pAtaA) cells at various ionic strengths directly reflect the properties of the AtaA molecule.

### Cell detachment and reversible immobilization using AtaA

**Fig. 3:**
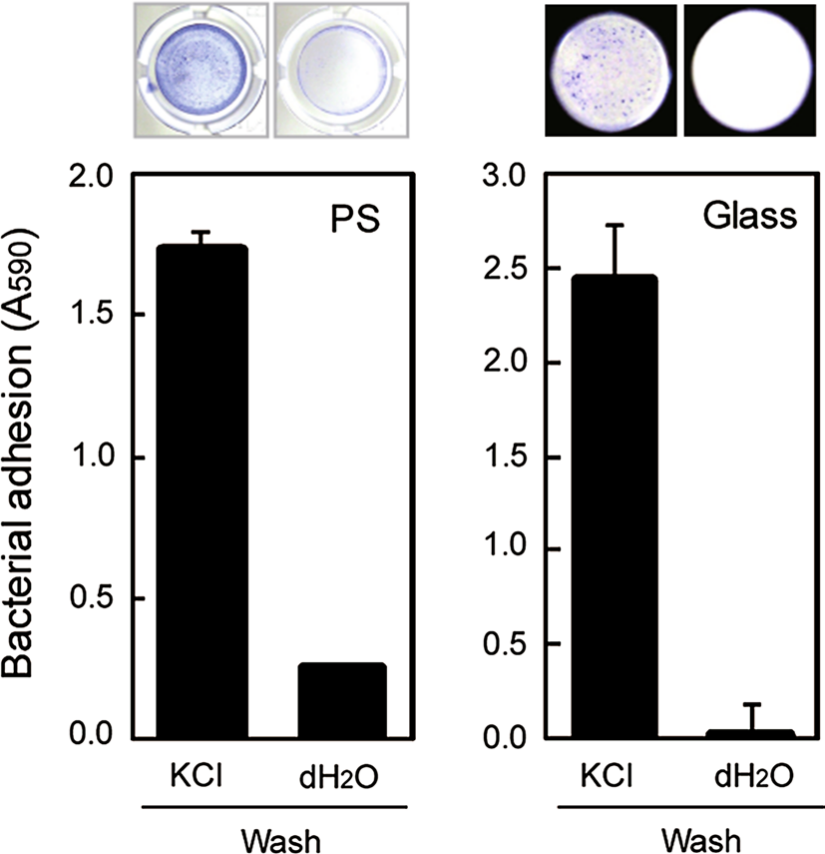
Detachment of ADP1 (pAtaA) cells immobilized on PS and glass surfaces. The cells retained on the surfaces after being washed with 100 mM KCl solution or dH_2_O were visualized and quantified by crystal violet staining. *Photographs* indicate the stained cells retained on the surface. Data are expressed as mean ± SEM (n = 3)

Our finding that the cell adhesion mediated by AtaA is inhibited by dH_2_O prompted us to examine the ability of a simple dH_2_O wash to detach bacterial cells already immobilized on material surfaces. After immobilization of ADP1 (pAtaA) cells onto the well surfaces in 100 mM KCl solution as described above, the wells were rinsed with 200 μL of dH_2_O or 100 mM KCl solution using a micropipette. This washing step was repeated three times. Thereafter, the cells still immobilized on the well surfaces were quantified by crystal violet staining. Most of the cells washed with dH_2_O were detached from both PS and glass surfaces, whereas the cells washed with 100 mM KCl solution were retained on the surfaces (Fig. 3).

We also confirmed the ability of dH_2_O to detach ADP1 (pAtaA) cells previously immobilized on a polyurethane foam support, which is often used in bioprocesses as a support. The cells were immobilized onto 1-cm^3^ pieces of polyurethane foam support. A piece of the support with the immobilized cells was transferred into fresh 100 mM KCl solution, gently rinsed, picked up with tweezers, and shaken in dH_2_O or 100 mM KCl solution for video recording. When shaken in dH_2_O, the immobilized cells immediately began to detach and an increase in the turbidity of the surrounding H_2_O solution was observed. An additional movie file shows this in more detail (see Additional file 1). In contrast, the immobilized cells were not detached at all by being shaken in 100 mM KCl solution (see Additional file 2).

**Fig. 4:**
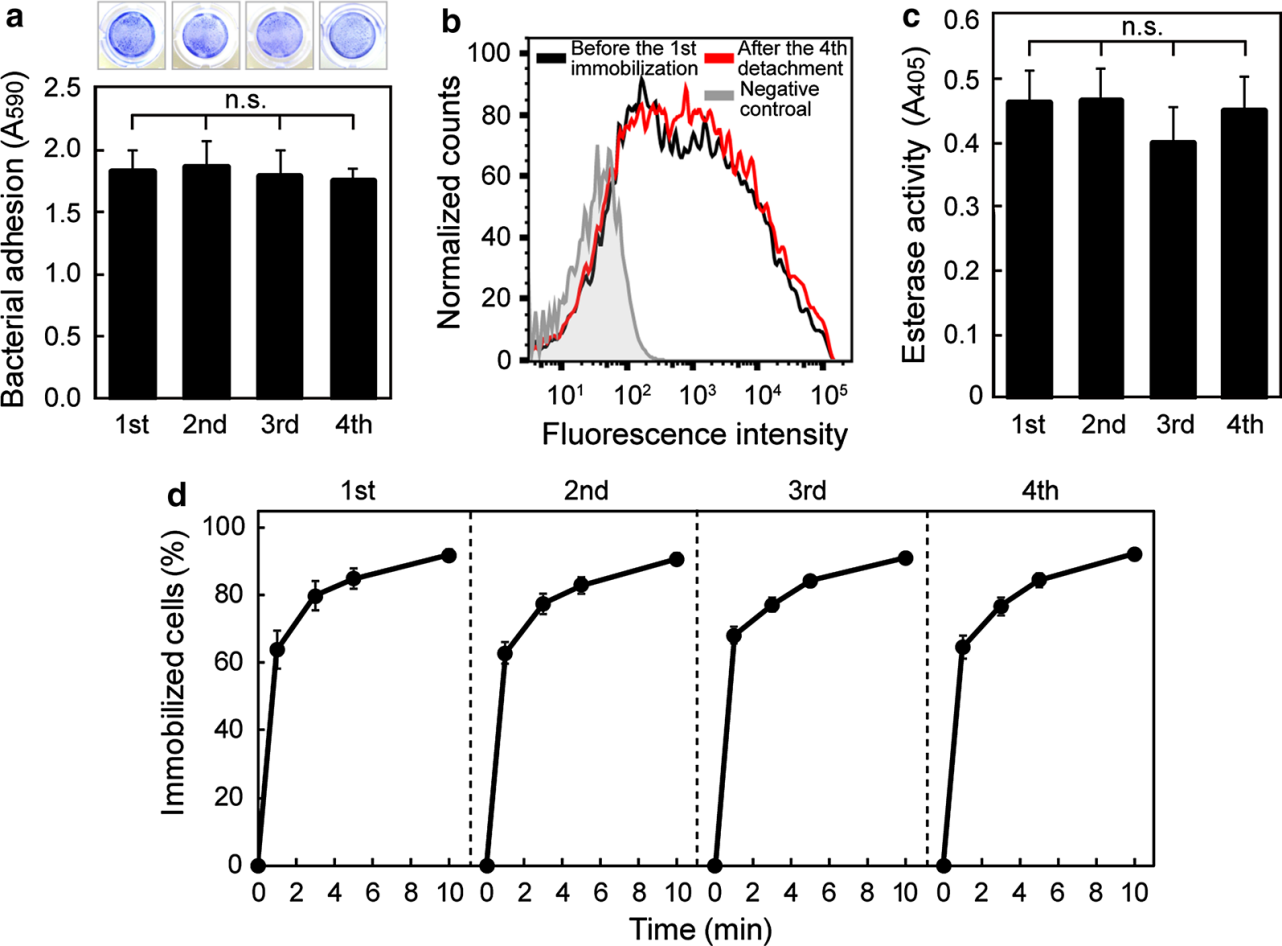
Re-immobilization of ADP1 (pAtaA) cells. **a** Repetition of the immobilization/detachment cycle of ADP1 (pAtaA) cells. The cells were immobilized onto well surfaces of a PS plate in 100 mM KCl solution for 2 h, detached by washing with dH_2_O from the PS plate, collected by centrifugation, resuspended in the KCl solution, added to fresh wells, and incubated for re-immobilization for 2 h. Immobilized cells were visualized and quantified by crystal violet staining. This process was repeated four times. *Photographs* indicate the stained cells immobilized on the surface at each cycle. **b** AtaA molecules on the ADP1 (pAtaA) cell surface before and after the repetition of the immobilization/detachment cycle were analyzed by flow cytometry. The *black* and *red lines* show surface-displayed AtaA before the first immobilization and after the fourth cycle of the repetition, respectively. The wild type of ADP1 without AtaA molecules was subjected to the same analysis as a negative control. **c** Esterase activities of ADP1 (pAtaA) cells immobilized on the plate wells in **a**. **d** Time course of the immobilization ratio of ADP1 (pAtaA) cells onto the polyurethane support in each immobilization/detachment cycle. In the respective cycles, the cells were immobilized onto polyurethane foam in 100 mM KCl solution in a flask with shaking and the immobilization ratio was calculated from a decrease in the OD_660_ of the cell suspension. Data are expressed as mean ± SEM (n = 3). *n.s.* not significant (ANOVA, *P* > 0.1)

Next, we tried to repeat the immobilization and detachment of bacterial cells expressing AtaA. ADP1 (pAtaA) cells were repeatedly subjected to the immobilization/detachment process; cells suspended in 100 mM KCl solution were immobilized onto the PS well surface by a 2-h incubation (immobilization process) and subsequently detached by the dH_2_O wash using the same procedure described above (detachment process). The detached cells were collected by centrifugation, resuspended in 100 mM KCl solution at an OD_660_ of 0.5, and placed into the new well for the next immobilization cycle. This immobilization/detachment process was repeated four times. As shown in Fig. 4a, the detached cells showed the same adhesion ability as the fresh cells used for the first adherence assay, and the cell adhesiveness did not decrease throughout four immobilization/detachment cycles. The cells finally detached were subjected to flow cytometry to quantify the amount of AtaA displayed on the ADP1 (pAtaA) cell surface. This revealed that the amount of AtaA molecules on the cell surface did not decrease even after the fourth detachment compared with that on fresh cells before the first immobilization (Fig. 4b), suggesting that AtaA was not impaired throughout the repeated detachment manipulations by washing with dH_2_O. The ADP1 (pAtaA) cells immobilized in each immobilization/detachment cycle were subjected to an esterase activity assay involving the addition of a reaction buffer containing a substrate directly to the well. The cell-bound esterase activity of the immobilized cells on the PS surface was maintained at the same level throughout the four cycles (Fig. 4c), suggesting that the repeated immobilization/detachment cycle also did not deteriorate the integrity of the surface of ADP1 (pAtaA) cells.

We also examined whether or not ADP1 (pAtaA) cells can be reversibly immobilized onto polyurethane foam support. Six pieces of the polyurethane foam (a 1 cm cube) were placed into a 30-mL cell suspension of ADP1 (pAtaA) at an OD_660_ of 2.0 in a 100-mL Erlenmeyer flask and shaken at 115 rpm for immobilization of the bacterial cells. The percentage of immobilized cells over time is shown in Fig. 4d (“1st”). More than 90% of the cells were immobilized within 10 min (immobilization process). Subsequently, the supernatant was discarded by decantation and 30 mL of dH_2_O was poured into the flask, which was then shaken at 115 rpm for 5 min. This washing step was repeated three times (detachment process). The detached cells from each washing step were collected by centrifugation and resuspended in 100 mM KCl solution at an OD_660_ of 2.0. Six fresh pieces of the polyurethane foam were placed into the cell suspension for the next immobilization cycle. This immobilization/detachment cycle was also repeated four times. The time profiles of the immobilization were similar throughout the four cycles; more than 90% of ADP1 (pAtaA) cells detached from the polyurethane were re-immobilized onto the fresh polyurethane foam within 10 min (Fig. 4d).

Thus, we have succeeded in developing a novel method for the reversible immobilization of bacterial cells, which enables the reuse of cells without impairment, by means of AtaA expression and a simple manipulation involving a dH_2_O wash. Other conventional immobilization methods are unsuitable for the development of a reversible process without support destruction or cell inactivation.

### Reuse of bacterial cells in a different type of reaction

**Fig. 5:**
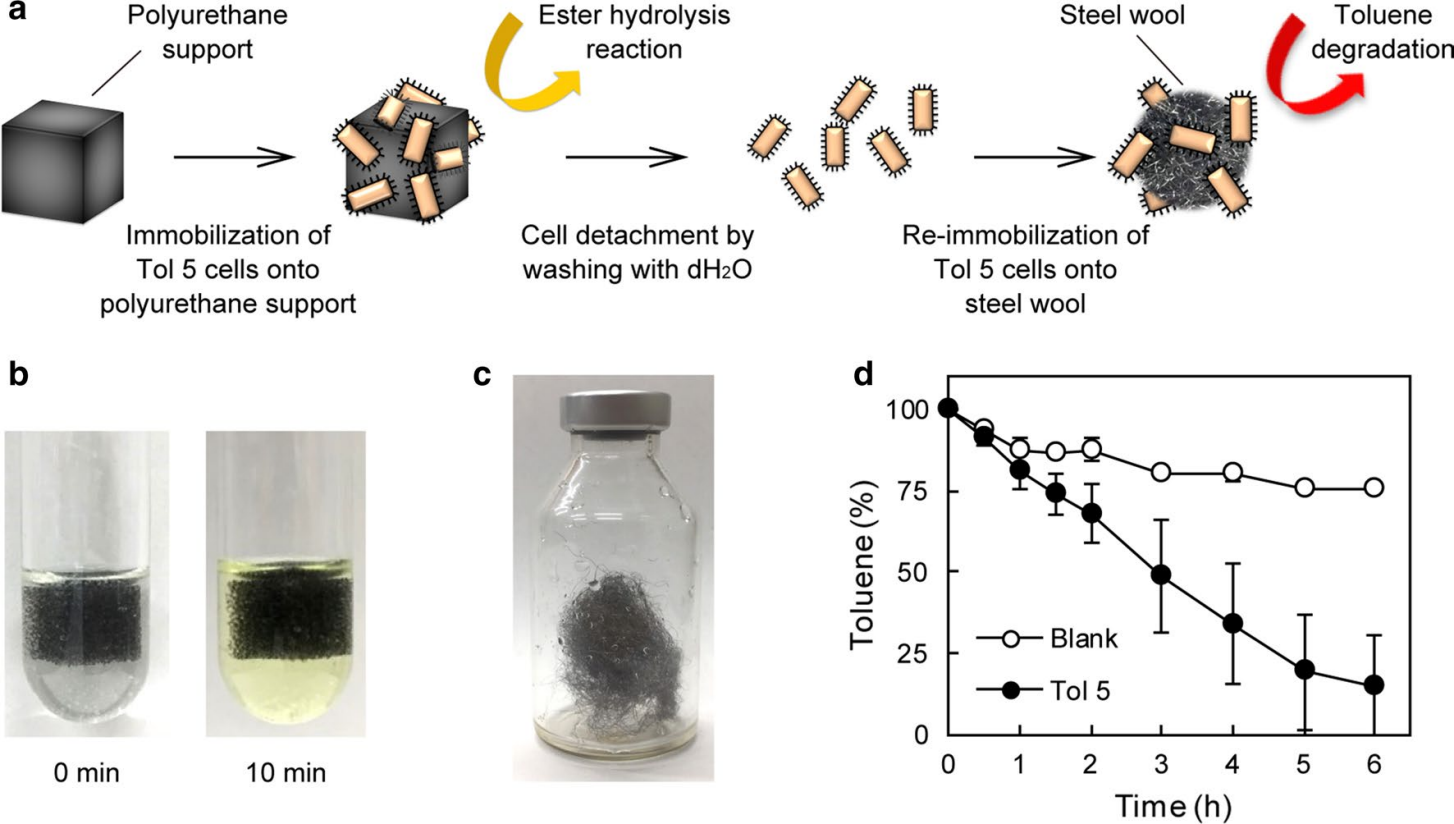
Reuse of bacterial cells for different reactions. **a** Model schema for the reuse of bacterial cells. **b** Ester hydrolysis reaction by immobilized Tol 5 cells. **c** Photograph of toluene degradation by Tol 5 cells re-immobilized on steel wool in a gas phase in a vial. **d** Time course of toluene degradation. Data are expressed as mean ± SEM (n = 3)

To show the merit of our reversible immobilization method, we attempted to demonstrate the reusability of bacterial cells for different types of reactions, an ester hydrolysis and toluene degradation, using the schema shown in Fig. 5a. At first, Tol 5 cells were immobilized onto three pieces of the polyurethane foam support in a 100-mL Erlenmeyer flask containing the cells suspended in 30 mL of 100 mM KCl solution (OD_660_ = 1.0) by shaking at 115 rpm at 28 °C for 30 min. The cells loosely attaching to the support were removed by dipping them into 100 mM KCl solution and gentle squeezing. A piece of the support with the immobilized Tol 5 cells was placed into esterase reaction buffer in a test tube. After a 10-min incubation of the test tube at 28 °C, the reaction buffer turned from colorless to yellow due to the 4-nitrophenol produced by esterase on the immobilized Tol 5 cells (Fig. 5b). Next, three pieces of the support used in the esterase reaction were collected and washed in 100 mL of dH_2_O in a 500-mL Erlenmeyer flask by shaking at 115 rpm for 5 min. This washing step was repeated three times. The detached cells from each washing step were collected by centrifugation, resuspended in 30 mL of basal salt (BS) medium in a 100-mL Erlenmeyer flask, and re-immobilized onto 300 mg of steel wool support, which is not eroded by organic solvents, by shaking at 115 rpm for 1 h.

For the induction of toluene-degrading gene expression, the steel wool support with the re-immobilized Tol 5 cells was picked up, touched with paper towel to remove extra water, transferred into a 25-mL vial, and incubated at 28 °C for 1 day under a toluene atmosphere. After this induction step, the immobilized cells on the steel wool support were subjected to a toluene-degradation reaction in a gas phase (Fig. 5c). The reaction was started by injecting 1 μL of toluene into the vial, and thereafter the toluene concertation of the gas in the vial was quantified by gas chromatography–mass spectrometry (GC/MS) and its time-dependent decrease was plotted (Fig. 5d). The cells immobilized on the steel wool support linearly degraded toluene for 5 h and thereafter the degradation rate lowered following the first-order reaction kinetics that depends on the toluene concentration. The slight decrease in the toluene concentration in the control vial without the bacterial cells (blank) suggests adsorption of toluene onto a butyl rubber septum on the cap or solubilization of toluene in a small amount of water from the wetting support. Thus, our reversible immobilization method uniquely allows us to reuse bacterial cells for different types of chemical reactions after detachment from a support, re-immobilization, and an appropriate induction or reactivation for second reaction.

### Reuse of supports for a different type of reaction

**Fig. 6:**
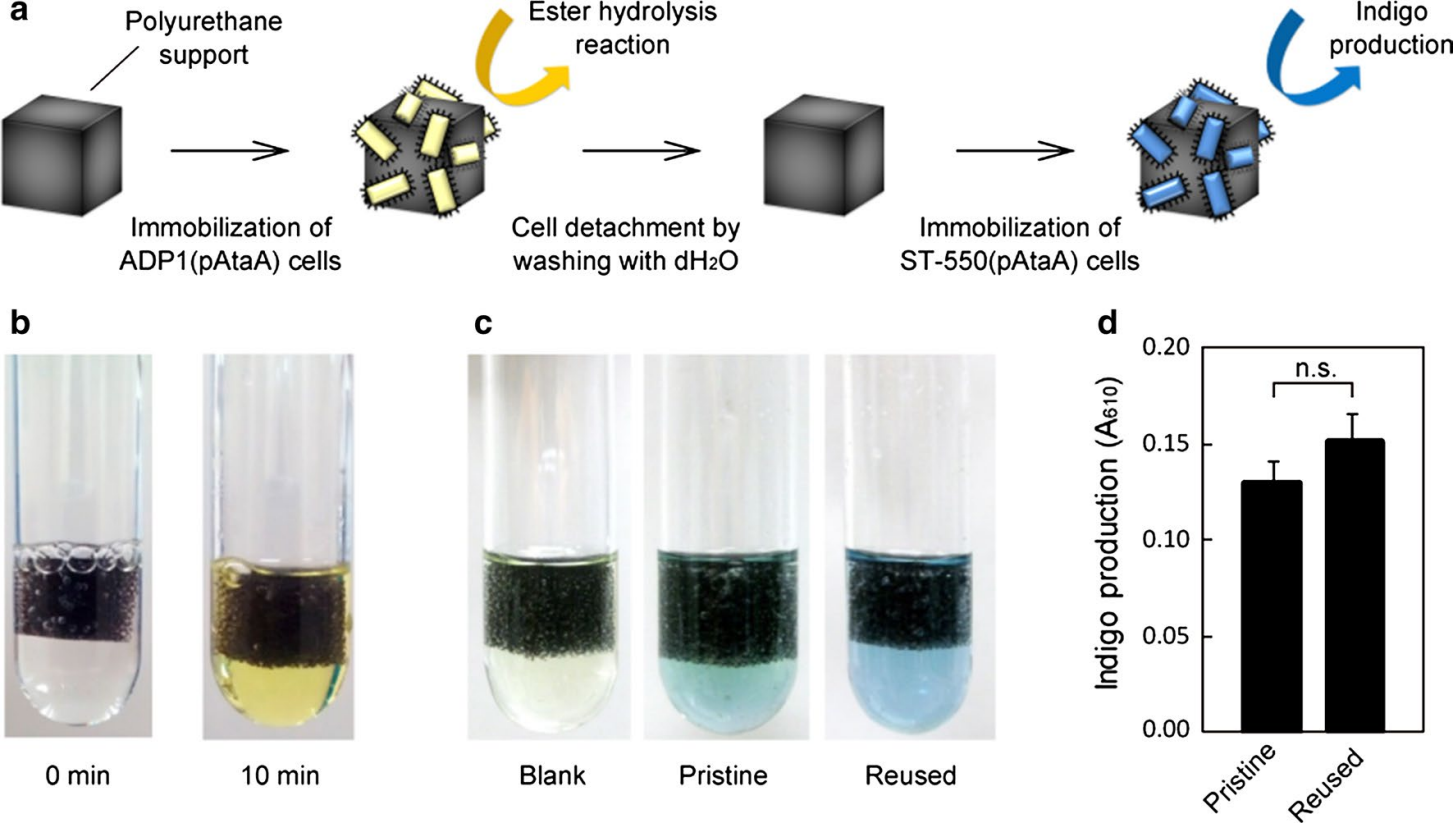
Reuse of supports for different reactions. **a** Model schema for the reuse of supports with two different bacterial strains. **b** Ester hydrolysis reaction by immobilized ADP1 (pAtaA) cells. **c** Indigo production by immobilized ST-550 (pAtaA) cells. **d** Quantification of indigo production by ST-550 (pAtaA) cells immobilized onto pristine or reused polyurethane supports in **c**. Data are expressed as mean ± SEM (n = 3). *n.s.* not significant (*t* test, *P* > 0.1)

To show the further merit of our reversible immobilization method, we attempted to demonstrate the reusability of the support used for the cell immobilization using the schema shown in Fig. 6a. Three pieces of the polyurethane foam support were placed into 30 mL of the suspension of resting ADP1 (pAtaA) cells in 100 mM KCl solution (OD_660_ = 1.0) and shaken in a 100-mL Erlenmeyer flask at 115 rpm at 30 °C for 30 min. The cells loosely attaching to the support were removed by dipping them into 100 mM KCl solution and gentle squeezing. A piece of the support with the immobilized ADP1 (pAtaA) cells was placed into an esterase reaction buffer in a test tube. After a 10-min incubation of the test tube at 28 °C, the reaction buffer turned from colorless to yellow due to the 4-nitrophenol produced by esterase on the immobilized ADP1 (pAtaA) cells, as with the Tol 5 cells (Fig. 6b).

Three pieces of the support used in the reaction were collected and washed in 100 mL of dH_2_O in a 500-mL Erlenmeyer flask by shaking at 115 rpm for 10 min. This washing step was repeated three times to thoroughly remove ADP1 (pAtaA) cells. Subsequently, three pieces of the used support were transferred into 30 mL of a suspension of resting cells of *Acinetobacter* sp. ST-550 transformed with *ataA*, ST-550 (pAtaA) [22], which has the ability to produce indigo from indole using its phenol hydroxylase [29, 30], in 100 mM KCl solution (OD_660_ = 1.0) and shaken in a 100-mL Erlenmeyer flask at 115 rpm at 30 °C for 30 min for cell immobilization. Pristine pieces of the polyurethane foam support were also subjected to the cell immobilization for a control experiment. The cells loosely attaching to the support were removed by dipping them into 100 mM KCl solution and gentle squeezing. Each piece of the support with the immobilized ST-550 (pAtaA) cells was placed into an indigo reaction solution and incubated for 12 h. Indigo produced by the immobilized ST-550 (pAtaA) was extracted with *N*,*N*-dimethylformamide (DMF). Figure 6c shows the solution extracted from each support. The quantity of indigo produced from each support is shown in Fig. 6d. The productivity with the reused support was similar to that with the pristine support, implying that the reused support retained its capability for bacterial immobilization after the immobilization/detachment process. Thus, our reversible immobilization method uniquely allows us to reuse supports, even for different chemical reactions, via the immobilization of different bacterial cells.

## Discussion

Immobilization of biocatalysts simplifies product separation, stabilizes biocatalysts, and enables the repeated or continuous use of biocatalysts, which are typically expensive to produce [12, 13]. However, they are usually discarded, together with the supports, after a reaction. In protein immobilization, many techniques for reversible immobilization of enzymes (e.g. lipase, amyloglucosidase, glucoamylase, and aminoacylase) have been studied to enable the regeneration and reuse of support materials [31–35]. Additionally, with regard to bacterial cell immobilization, reversibility should be beneficial. However, the reuse of gel supports is impossible after their use in entrapment immobilization, which is most frequently employed for bacterial cells. Biofilm reactors are also used in the production of valuable compounds, such as alcohols and organic acids, not just in wastewater treatment [15, 36, 37]. These bioreactors use biofilms formed on support materials as immobilized microbial cells [15, 38]. Once biofilms are formed, it is difficult to completely detach them from supports by simple treatments. Therefore, when catalytic activity decreases or a chemical reaction has to be switched for another one, the support with biofilms would be discarded and a new biofilm would be reconstructed on the fresh support. However, a long startup time is required to rebuild an active biofilm.

In this study, we found that the stickiness of the AtaA molecule is drastically diminished at a lower ionic strength and is completely lost in dH_2_O (Fig. 1). Cell adhesion mediated by AtaA also depends on ionic strength in the same manner as the AtaA molecule, and even bacterial cells previously adhered to supports through AtaA can be detached in dH_2_O (Figs. 2, 3). Based on this adhesion property, we have established a reversible immobilization method for microbial cells (Fig. 4) and demonstrated the reuse of both cells and supports by means of this reversible immobilization (Figs. 5, 6).

Cells immobilized with AtaA can be detached from supports by a simple manipulation involving a dH_2_O wash and active cells can be quickly immobilized onto the same previously used support. Because this method is not based on the characteristics of support materials but the unique adhesion property of AtaA, various materials can be employed as reusable supports. For example, supports that have a structure with pores, fibers, or slits for a large surface area and are formed in a combined unit or integrated into a reactor vessel might be used.

In our new reversible immobilization method, both cells and supports can be reused for different types of chemical reactions. Three patterns can be considered about reused processes; (1) used cells are re-immobilized onto a new support, (2) fresh cells are immobilized onto a used support, and (3) used cells are re-immobilized onto a used support. In other words, one of or both of cells and a support are reused. It is expensive to grow bacterial cells on a medium containing many kinds of chemicals, such as nutrients, inducers, and antibiotics, using energy for sterilization, agitation, aeration, and temperature control. In this study, we demonstrated that bacterial cells can be reused for a different type of chemical reaction after a simple induction or reactivation step. We can choose a different support material that is suitable for the subsequent reaction. For example, we used polyurethane foam for the first reaction of ester hydrolysis in a buffer and steel wool for the second reaction of toluene degradation in a gas phase. In addition, the polyurethane foam can also be reused for a different reaction, such as indigo production, after immobilization of a different bacterial strain. The reuse of support material mitigates problems caused by the use of support materials, such as the cost and waste of support materials. Reversible microbial cell immobilization would make bioreactors and bioprocesses simpler, more efficient, more cost-effective, and more convenient.

## Conclusions

In summary, we found that the stickiness of isolated AtaA PSD and cell adhesion mediated by AtaA are drastically diminished in deionized water and that deionized water even detaches bacterial cells previously adhered to support in a salt solution. Using this phenomenon, we invented a unique reversible immobilization method that allows us to reuse bacterial cells and supports for different chemical reactions by a simple manipulation involving a dH_2_O wash. This method for the immobilization of bacterial cells using AtaA would make bioprocesses more cost-effective and enhance their commercial use for environmentally friendly chemical productions.

## Methods

### Bacterial strains and culture conditions

The bacterial strains used in this study are detailed in Table 1. These bacterial strains were grown as described previously [20].

**Table 1:** Bacterial strains and plasmids used in this study

Strains or plasmids	Description	Reference
*Acinetobacter*		
sp. Tol 5	Wild type strain	[21]
sp. Tol 5 4140	Unmarked Δ*ataA* mutant of Tol 5	[27]
sp. Tol 5 4140 (p3CAtaA)	Tol 5 4140 harboring p3CAtaA plasmid	[26]
*baylyi* ADP1	Wild type strain: ATCC 33305	[40]
*baylyi* ADP1 (pAtaA)	ADP1 harboring pAtaA plasmid	[20]
sp. ST-550	Indigo productive strain	[30]
sp. ST-550 (pAtaA)	ST-550 harboring pAtaA plasmid	[22]
Plasmids		
pARP3	*E. coli*-*Acinetobacter* shuttle expression vector, *araC*-P_*BAD*_, Gm^r^, Amp^r^	[20]
pAtaA	*ataA*-expression vector, pARP3::*ataA*	[20]
p3CAtaA	*3CataA*-expression vector, pARP3::*3CataA*	[26]

### Enzyme-linked immunosorbent assay (ELISA)

AtaA PSD was isolated and purified from the Tol 5 derivative strain 4140 [27] harboring p3CAtaA plasmid (Tol 5 4140 (p3CAtaA)), as described previously [26]. Twenty-five microliters of the purified AtaA PSD (10 μg/mL) and an equal volume of KCl solution were added to each well of 96-well PS plates (353072; Becton, Dickinson and Company, NJ) or 96-well glass plates (FB-96; Nippon Sheet Glass Company, Ltd., Tokyo, Japan) and incubated at 28 °C for adsorption. After a 2-h incubation, the solution was removed by a micropipette and each well was gently rinsed three times by removing and replacing 200 μL of PBS-T. Then, 200 µL of PBS-T containing 2% skim milk was added to each well and incubated for 30 min for blocking. Thereafter, the protein adsorbed to the well surface was treated with anti-AtaA_699–1014_ antiserum [20] at a 1:10,000 dilution in PBS-T for 30 min and subsequently with a HRP-conjugated anti-rabbit antibody at a 1:10,000 dilution in PBS-T for 30 min. Finally, the wells were rinsed five times with PBS-T, and 100 μL of substrate solution [ELISA POD Substrate TMB Solution (Popular); Nacalai Tesque, Kyoto, Japan] was added, followed by a 15-min incubation at room temperature. The reaction was stopped by the addition of 100 μL of 1.0 M H_2_SO_4_ and the absorbance at 450 nm (A_450_) was measured using a microplate reader (ARVO X3; PerkinElmer, Inc., MA).

### Quartz crystal microbalance

The adhesiveness of AtaA PSD was measured using a QCM system (AFFINIX Q8; ULVAC, Kanagawa, Japan) as described previously [26] with a slight modification. A gold-coated electrode (QCM01S-01; ULVAC) was cleaned with piranha solution (H_2_SO_4_:30% H_2_O_2_ = 3:1) and equilibrated with 98 μL of dH_2_O at 25 °C. After the equilibration, 1 μL of AtaA PSD solution (0.1 mg/mL) was added and the frequency change was measured for 5 min. Subsequently, 1 μL of 100 mM KCl solution (final concentration = 1 mM) was added and the measurement was continued.

### Cell attachment and detachment assays using well plates

For a cell attachment assay, the harvested cells were washed three times with dH_2_O and suspended in KCl solutions of various concentrations or dH_2_O at an OD_660_ of 0.5. The cell suspensions (200 μL each) were placed into wells of 96-well PS or glass plates. After a 2-h incubation at 28 °C without shaking, the cell suspensions were removed by a micropipette and each well was rinsed three times with 200 μL of KCl solution of each concentration or dH_2_O. The immobilized cells were stained with 200 μL of 0.1% crystal violet solution for 15 min. After three rinses with 200 μL of KCl solution of each concentration or dH_2_O, the dye was eluted from the cells with 200 μL of 70% ethanol, and the absorbance at 590 nm (A_590_) of the elution was measured by a microplate reader.

For a cell detachment assay, each well was rinsed three times with 200 µL of 100 mM KCl solution or dH_2_O using a micropipette. The remaining cells were quantified by crystal violet staining as described above. For re-attachment, the cells detached by rinsing each well with dH_2_O were collected by centrifugation, resuspended in 100 mM KCl solution at an OD_660_ of 0.5, and added to a new well for the next immobilization cycle.

### Immobilization of bacterial cells onto support materials

Polyurethane foam with a specific surface area of 37.5 cm^2^/cm^3^ (CFH-30; Inoac Corporation, Nagoya, Japan) in the shape of a cube (1 cm^3^) was used as a sponge support. The steel wool used in this study was the same as that previously used [25], which was purchased from Handy Crown (Tokyo, Japan).

To immobilize bacterial cells onto the polyurethane foam support, cells were suspended in 100 mM KCl solution in a 100-mL Erlenmeyer flask. The value of the OD_660_ was adjusted to 2.0 for the visualization of the cell detachment from the support and for the analysis of the time profile of cell immobilization or to 1.0 for use in chemical reactions. Pieces of the support were placed into the cell suspension and shaken at 115 rpm at 28 or 30 °C for 10–30 min. For analysis of the time profile of cell immobilization, the OD_660_ of the cell suspension was measured periodically. The immobilization ratio of the cells was calculated from the following equation:

$${\text{Immobilization ratio }}\left( \% \right) = {{\left( {{\text{OD}}_{{660\;{\text{initial}}}} - {\text{OD}}_{{660\;{\text{after shaking}}}} } \right)} \mathord{\left/ {\vphantom {{\left( {{\text{OD}}_{{660\;{\text{initial}}}} - {\text{OD}}_{{660\;{\text{after shaking}}}} } \right)} {{\text{OD}}_{{660\;{\text{initial}}}} \times 100.}}} \right. \kern-0pt} {{\text{OD}}_{{660\;{\text{initial}}}} \times 100.}}$$

To immobilize bacterial cells onto the steel wool support, the cells detached from the three pieces of the polyurethane foam support used for the esterase reaction were resuspended in 30 mL of BS medium [39] in a 100-mL Erlenmeyer flask. Into this cell suspension, 300 mg of the steel wool support was placed and shaken at 115 rpm at 28 °C for 1 h.

### Flow cytometry

Bacterial cells before and after the cell immobilization/detachment test were resuspended in PBS containing 4% paraformaldehyde and incubated at room temperature for 15 min. The samples were washed with PBS and treated with anti-AtaA_699–1014_ antiserum diluted 1:10,000 in PBS. After a 1-h incubation at room temperature, the samples were washed twice with NET buffer (150 mM NaCl, 5 mM EDTA, 50 mM Tris–HCl, 0.05% Triton X-100, pH 7.6) and treated with Alexa Fluor 488-conjugated anti-rabbit antibody (Cell Signaling Technology, MA) diluted 1:500 in NET buffer for 30 min. Finally, the samples were resuspended in dH_2_O, and the fluorescence was measured by FACS Canto II (Becton, Dickinson and Company, NJ).

### Chemical reactions by immobilized bacteria

For the measurement of cell-bound esterase activity, cells immobilized on plate wells were reacted with 1.9 mM 4-nitrophenyl butyrate (4-NPB) in 200 μL reaction buffer (1.1% Triton X-100, 50 mM 3,3-dimethylglutaric acid, 50 mM Tris, 50 mM 2-amino-2-methyl-1,3-propanediol) at 28 °C for 30 min. Triton X-100 was eliminated from the reaction buffer when the esterase activity of Tol 5 was measured. The A_405_ of 4-nitrophenol produced by the reaction was measured by a microplate reader. To measure the esterase activity of cells immobilized on the polyurethane support, a piece of the support with the immobilized cells was placed into 3 mL of the reaction buffer in a test tube and incubated at 28 °C for 10 min.

For indigo production, a piece of polyurethane foam support with immobilized cells was transferred into a 3-mL reaction solution (1 mM indole, 1% DMF, 40 mM potassium phosphate buffer, pH 7.0) in a test tube and incubated at 30 °C for 12 h. Indigo produced from indole was extracted with DMF, and its concentration was determined using A_610_ measurement.

For toluene degradation, bacterial cells immobilized on steel wool were placed in a 25-mL vial capped by a butyl rubber septum. The reaction was started by injecting 1 μL of toluene into the vial using a gastight syringe (MS-GFN100; ITO Corporation, Fuji, Japan). The toluene concentration was measured using GC/MS, which comprised a GC system (GC7820A; Agilent Technologies, Santa Clara, CA) coupled to a MS detector (MSD 5975; Agilent Technologies), equipped with a Rtx-200 capillary column (30 m × 0.32 mm × 0.5 µm; RESTEC, Bellefonte, PA). Gas sample (50 µL) was taken from a vial and injected into the GC/MS system using the gastight syringe. The split ratio and the flow rate of helium were set at 10:1 and 2 mL/min, respectively. The operating program started with an isocratic step at 90 °C for 1 min, followed by temperature ramping of 25 °C/min to the final temperature at 120 °C, then ion fragments of m/z = 65 and m/z = 91 were monitored on the selected ion monitoring mode. The peak, which showed toluene, was detected at a retention time of 1.4 min. The degradation ratio of toluene was calculated from the ratio of the peak area at each time to that at 0 min.

## Additional files

**Additional file 1.** Detachment of adhered ADP1 (pAtaA) cells from a polyurethane support by washing in dH_2_O. The polyurethane supports were put into the ADP1 (pAtaA) cell suspension in 100 mM KCl solution and were incubated at 28 °C with shaking at 115 rpm. After incubation for 10 min, the supports were retrieved and lightly washed with 100 mM KCl solution. Then, the supports were picked up with tweezers and shaken in 100 mL of dH_2_O for 1 min. Note that the white grains in the dH_2_O are detached cells.

**Additional file 2.** Detachment of adhered ADP1 (pAtaA) cells from a polyurethane support by washing in 100 mM KCl solution. The polyurethane supports were put into the ADP1 (pAtaA) cell suspension in 100 mM KCl solution and were incubated at 28 °C with shaking at 115 rpm. After incubation for 10 min, the supports were retrieved and lightly washed with 100 mM KCl solution. Then, the supports were picked up with tweezers and shaken in 100 mL of 100 mM KCl solution for 1 min.

## Abbreviations

TAA: trimeric autotransporter adhesion
PSD: passenger domain
TM: transmembrane anchor
HRV 3C: human rhinovirus 3C
dH_2_O: deionized water
ELISA: enzyme-linked immunosorbent assay
HRP: horseradish peroxidase
POD: peroxidase
QCM: quartz crystal microbalance
4-NPB: 4-nitrophenyl butyrate
BS: basal salt
GC/MS: gas chromatography–mass spectrometry
DMF: *N*,*N*-dimethylformamide
PS: polystyrene
